# Transformation-Induced Relaxation and Stress Recovery of TiNi Shape Memory Alloy

**DOI:** 10.3390/ma7031912

**Published:** 2014-03-06

**Authors:** Kohei Takeda, Ryosuke Matsui, Hisaaki Tobushi, Elzbieta A. Pieczyska

**Affiliations:** 1 Department of Mechanical Engineering, Aichi Institute of Technology, 1247 Yachigusa, Yakusa-cho, Toyota 470-0392, Japan; E-Mails: r_matsui@aitech.ac.jp (R.M.); tobushi@aitech.ac.jp (H.T.); 2 Institute of Fundamental Technological Research, Polish Academy of Sciences, Pawinskiego 5B, Warszawa 02-106, Poland; E-Mail: epiecz@ippt.pan.pl

**Keywords:** shape memory alloy, superelasticity, titanium-nickel alloy, subloop, stress relaxation, stress recovery, martensitic transformation

## Abstract

The transformation-induced stress relaxation and stress recovery of TiNi shape memory alloy (SMA) in stress-controlled subloop loading were investigated based on the local variation in temperature and transformation band on the surface of the tape in the tension test. The results obtained are summarized as follows. (1) In the loading process, temperature increases due to the exothermic martensitic transformation (MT) until the holding strain and thereafter temperature decreases while holding the strain constant, resulting in stress relaxation due to the MT; (2) In the unloading process, temperature decreases due to the endothermic reverse transformation until the holding strain and thereafter temperature increases while holding the strain constant, resulting in stress recovery due to the reverse transformation; (3) Stress varies markedly in the initial stage followed by gradual change while holding the strain constant; (4) If the stress rate is high until the holding strain in the loading and unloading processes, both stress relaxation and stress recovery are large; (5) It is important to take into account this behavior in the design of SMA elements, since the force of SMA elements varies even if the atmospheric temperature is kept constant.

## 1. Introduction

Since shape memory alloy (SMA) shows shape memory effect and superelasticity (SE) and has superior functions as an intelligent material, the application of the SMA has drawn worldwide attention [1–3]. In order to design shape memory elements in the application of SMA, the thermomechanical properties of the material are important. The functional properties of SMA appear mainly based on the martensitic transformation (MT). Since the MT of the SMA depends on temperature, stress and their hysteresis, the deformation properties due to the MT are complex [4–6]. Most studies on the thermomechanical properties have been carried out until now under the condition of a full or perfect loop in which the MT and reverse transformation complete. In practical applications, strain, temperature and stress vary in various ranges. In the case of the subloop, partial loop, or inner loop in which strain, temperature, and stress vary in the range prior to the MT completion, the starting and finishing conditions of the MT prescribed in the full loop are not satisfied. The progress of the MT and reverse transformation therefore changes depending on the hysteresis of strain, temperature and stress [7–9]. For example, two-way deformation appears in the SMA element due to the MT and reverse transformation by heating and cooling under constant stress. In the case of the full loop, the SMA element elongates and shrinks by the amount of deformation or stroke corresponding to the available MT strain. However, in the case of subloop, the stroke corresponding to the MT strain cannot be obtained in the SMA element. In a similar way, since the recovery stress which appears by heating and cooling under constant strain varies depending on the hysteresis, the recovery stress in the subloop becomes smaller than that in the full loop. Therefore, when the recovery stress is used as the driving force of actuator or robot, the recovery stress in the subloop cannot be used effectively, as in the full loop. In addition, since the MT depends on the rates of loading-unloading and heating-cooling, the variations in strain and stress are complex.

If the SMA is subjected to subloop loading, the transformation-induced creep appears under constant stress [10] and the transformation-induced stress relaxation occurs under constant strain [9]. In this paper, although variation in stress was considered to be induced due to either an exothermic or endothermic reaction, the variation in temperature distribution on the specimen surface was not observed directly. It has not been observed up to the present since the amount of progress of the MT is small under constant strain and therefore the observation of the transformation progressing process is not easy. In practical applications of SMA like in a clutch or a brake to control the motion of machines, in an actuator to control the driving force by oil pressure and in a tightening element, it is necessary to control the load. If SMA is used in these devices, it is necessary to understand the deformation behavior in the stress-controlled condition. In many cases of these SMA applications, the element is controlled to operate in a certain position and thereafter to keep the position. If the condition of the MT to progress is satisfied, stress relaxation and stress recovery occur under constant strain and the load to hold the position varies. Similarly, even if the load is controlled to remain constant in order to hold the position, creep and creep recovery may occur under constant stress, resulting in difficulty in controlling the position. Therefore, considering the control of load and position by using the SMA, it is necessary to understand the stress-strain-temperature relationship of the SMA. In order to evaluate the functional properties of SMA elements subjected to subloop loading and to design the SMA elements for practical use, the deformation behavior in the subloop loading is very important.

In the present study, transformation-induced relaxation and stress recovery in the stress-controlled SE subloop loading are discussed with a tension test for TiNi SMA, which is most widely used in practical applications. If it is loaded under a constant stress rate and thereafter strain is held constant in the MT region, stress relaxation appears. If strain is held constant in the reverse transformation region during unloading, stress recovery appears. Although phase transformation front propagation has been investigated in full loop loading under a constant strain rate or stress rate [11,12], phase transformation front propagation has been reported little in subloop loading. In particular, since front propagation under a constant strain is very small and not easy to observe, it has not been reported up until now. Based on the changes in the transformation band observed by microscope and the temperature distribution by thermography on the specimen surface in the experiment, the characteristics of stress relaxation and stress recovery have been clarified. The influence of stress rate on the stress relaxation and stress recovery is also discussed.

## 2. Deformation Properties in Subloop Strain-Controlled and Stress-Controlled Loadings

**Figure 1.**
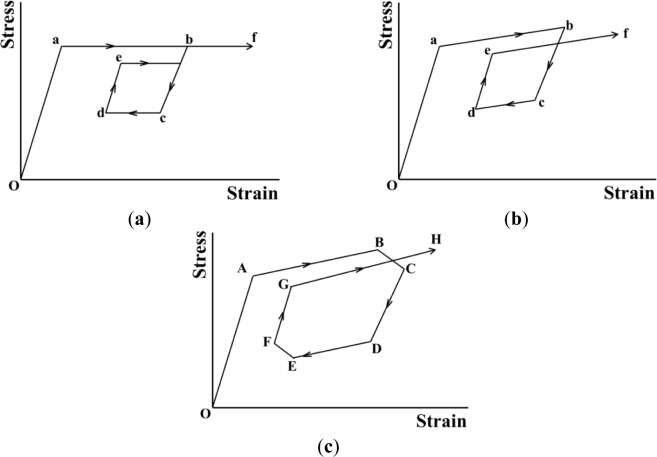
Stress-strain diagrams in superelasticity (SE) subloop strain-controlled and stress-controlled loadings under various loading rates. (**a**) Constant low strain rate; (b) Constant high strain rate; (**c**) Constant stress rate.

The SE stress-strain diagrams in subloop strain-controlled and stress-controlled loadings under a constant low strain rate, high strain rate and stress rate are schematically shown in Figure 1a–c, respectively [9,13]. As shown in Figure 1a, in the case of subloop unloading from a point b and reloading from a point d under a constant low strain rate, the second upper stress plateau during reloading appears due to the MT at a point e under a little lower stress than that in the first stress plateau and thereafter the curve passes through the unloading start point b, showing the return-point memory [5,9,13].

As shown in Figure 1b, in the case of subloop loading and unloading under a constant high strain rate, temperature increases due to the exothermic stress-induced martensitic transformation (SIMT) and stress increases in the upper stress plateau. If it is reloaded from a point d following unloading from a point b, the SIMT starts at a point e and the reloading curve does not pass through the point b. The return-point memory therefore does not appear.

As shown in Figure 1c, in the case of subloop loading and unloading under a constant stress rate, stress increases in the loading process from a point A to a point B since temperature increases due to the SIMT. In the unloading process from the point B, the material is cooled by the air, resulting in a decrease in temperature. The condition for the MT to progress therefore is satisfied in the unloading process from point B to a point C, and strain increases with accompaniment of an overshoot of strain. Temperature decreases due to the reverse transformation in the unloading process from a point D to a point E, and temperature increases due to heating by the air in the reloading process from point E to a point F. The condition for the reverse transformation to progress therefore is satisfied from point E to point F, and strain decreases with accompaniment of an undershoot of strain [9]. The following reloading curve from a point G to a point H in the transformation region does not pass through the unloading start point B and the return-point memory therefore does not appear.

As mentioned above, in stress-controlled subloop loading, overshoot and undershoot of strain appear in the early stage of unloading and reloading and do not appear in strain-controlled subloop loading. Therefore, in order to design SMA elements which control force, it is important to understand the deformation behavior in the stress-controlled subloop loading.

## 3. Experimental Method

### 3.1. Materials and Specimen

**Figure 2.**
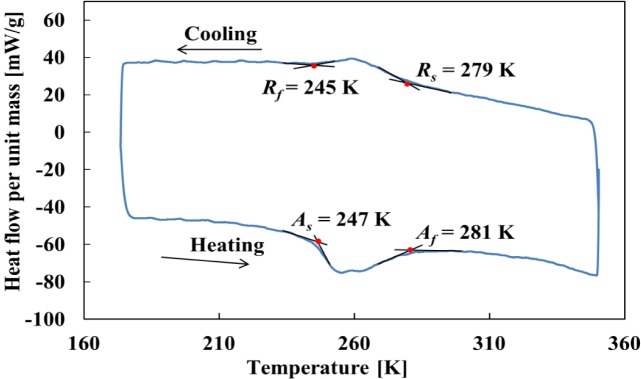
Differential scanning calorimetry (DSC) thermogram for material used in the experiments.

The material was a Ti-50.95at%Ni SMA tape to show SE at room temperature, produced by Furukawa Techno Material, Co., Ltd. (Hiratsuka, Japan). Thickness of the material was 0.37 mm, width 9.95 mm and length 170 mm. The material was held at 803 K for 1 min in the shape-memory heat treatment process. The differential scanning calorimetry (DSC) (Shimadzu, Co., Kyoto, Japan) thermogram of the material is shown in Figure 2. The R-phase transformation start and finish temperatures *R_s_* and *R_f_*, and the reverse transformation start and finish temperatures *A_s_* and *A_f_* obtained by the DSC were 279 K, 245 K, 247 K and 281 K, respectively. The specimen was the tape with uniform shape. The gauge length was the distance of 100 mm between grippers. The specimen surface to observe the transformation band was mirror-finished with emery paper No. 2000. The specimen surface to observe the temperature distribution by thermography was covered with a uniform thin soot layer from a candle. In the process of covering the specimen surface with the soot, a small plate was used for the soot formation between the specimen and a candle. With this procedure, the specimen was kept away from the candle flame, and the influence of heat while covering the specimen surface with the soot was therefore slight.

### 3.2. Experimental Apparatus

The SMA-characteristic testing machine (EZ Graph, Shimadzu, Co., Kyoto, Japan) was used in the tension test. The transformation band on the specimen surface was observed by a motion analysis microscope (VW-6000, Keyence, Co., Osaka, Japan). The temperature variation due to the exothermic and endothermic MT on the specimen surface was observed by infrared thermography (Thermo Tracer H2600, Nippon Avionics, Co., Ltd., Tokyo, Japan).

### 3.3. Experimental Procedure

The tension test on the stress relaxation and stress recovery characteristics in the stress-controlledSE subloop loading was carried out at room temperature in air. The transformation band and temperature distribution on the specimen surface were observed during the test. The loading process took place under a constant stress rate dσ/d*t* until a certain strain ε_1_ and thereafter stress relaxation was observed by holding ε_1_ constant. The following unloading process was conducted under a constant stress rate dσ/d*t* until a certain strain ε_3_ and thereafter stress recovery was observed by holding ε_3_ constant. The stress rates dσ/d*t* were 1 MPa/s, 3 MPa/s and 5MPa/s. The holding strains ε_1_ and ε_3_ in the loading and unloading processes were 6% and 2%, respectively.

## 4. Experimental Results and Discussion

### 4.1. Stress relaxation and Stress Recovery

#### 4.1.1. Stress-Strain Relationship in Stress-Controlled Subloop Loading

**Figure 3.**
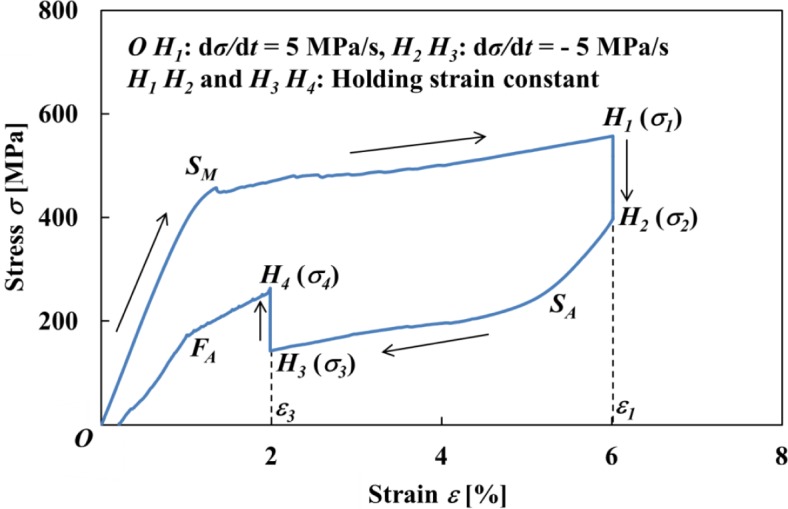
Stress-strain curve obtained by the test under a stress rate of dσ/d*t* = 5 MPa/s until a point *H*_1_ followed by holding strain constant at ε_1_ = 6% and thereafter unloading until a point *H*_3_ followed by holding the strain constant at ε_3_ = 2%.

**Figure 4.**
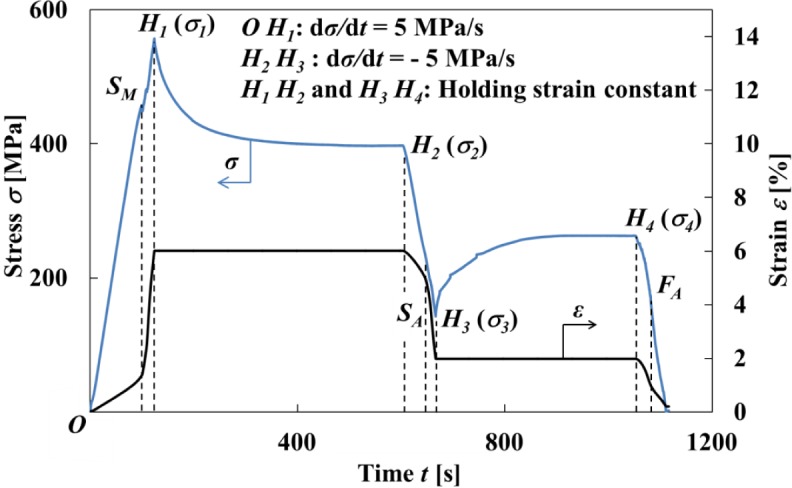
Variation in stress and strain with time obtained by the test under a stress rate of dσ/d*t* = 5 MPa/s until a point *H*_1_ followed by holding the strain constant until a point *H*_2_ and thereafter unloading until a point *H*_3_ followed by holding the strain constant until a point *H*_4_.

The stress-strain curve and variations in stress σ and strain ε with time *t* obtained by the test under a stress rate dσ/d*t* = 5 MPa/s until a point *H*_1_ at a strain ε_1_ = 6% followed by holding the strain ε_1_ constant and thereafter unloading under a stress rate dσ/d*t* = −5 MPa/s until a point *H*_3_ at a strain ε_3_ = 2% followed by holding the strain ε_3_ constant, are shown in Figures 3 and 4, respectively.

As can be seen in Figure 3, in the strain holding process at ε_1_ = 6% following loading till the strain ε_1_ under the stress rate dσ/d*t* = 5 MPa/s, stress decreases from σ_1_ to σ_2_, resulting in stress relaxation *∆*σ = σ_2_ − σ_1_. In the strain holding process at ε_3_ = 2% following unloading till the strain ε_3_ under the stress rate dσ/d*t* = −5 MPa/s, stress increases from σ_3_ to σ_4_, resulting in stress recovery *∆*σ = σ_4_ − σ_3_.

Stress fluctuates just after a MT starting point *S_M_* in the loading process as observed in Figures 3 and 4.

The transformation band occurs due to the SIMT at the point *S_M_*, and an overshoot of stress appears. Compared to a strain rate dε/d*t* = 1.37 × 10^−4^ s^−1^ until the point *S_M_*, the strain rate dε/d*t* increases suddenly to 1.94 × 10^−3^ s^−1^ after the point *S_M_*. This is caused by the fact that a cross-head in the tension test machine moves under higher velocity since it is being controlled to keep a constant stress rate, and an overshoot of stress therefore appears. In order to follow this sudden velocity change, first of all stress fluctuates just after the point *S_M_*. Then after the strain holding start point *H*_1_, stress decreases rapidly in the early stage and thereafter decreases gradually. The stress σ_2_ at a point *H*_2_ after relaxation is 397 MPa. Similarly, in the unloading process, since undershoot of stress appears, the strain rate dε/d*t* increases suddenly from −2.34 × 10^−4^ s^−1^ to −1.51 × 10^−3^ s^−1^ after the reverse transformation start point *S_A_*. After the strain holding start point *H*_3_, stress increases rapidly in the early stage and thereafter increases gradually. The stress σ_4_ at a point *H*_4_ after recovery is 263 MPa.

#### 4.1.2. Behavior of the Transformation Band

**Figure 5.**
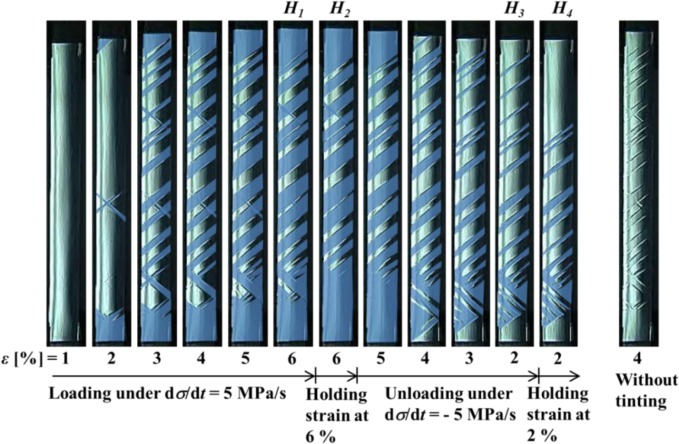
Photographs of specimen surface at various strains ε under a stress rate of 5 MPa/s followed by holding strain constant at a point *H*_1_ (ε_1_ = 6%) during loading and at a point *H*_3_ (ε_3_ = 2%) during unloading.

Figure 5 shows the photographs of the specimen surface at various strains taken by a microscope during the test which correspond to the stress-strain curve shown in Figure 3. The photograph on the right hand side shows the surface without tinting at a strain of 4% in the loading process. As can be seen from this photograph, although the interface of the transformation band can be observed, the region of the austenite phase (A-phase) and that of the martensitic phase (M-phase) are not clearly recognized at a glance. In order to show the region of the M-phase clearly, the M-phase is tinted by deep blue in Figure 5. In the loading process under a constant stress rate of 5 MPa/s, strain rate dε/d*t* increases from a strain of 2% to 3%, and the transformation bands appear on the whole specimen surface. After a strain of 3%, the interfaces of the appeared transformation bands propagate and strain increases. In the strain holding stage from the point *H*_1_ at a strain of 6% to the point *H*_2_, the MT progresses and the region of the M-phase expands a little. In the unloading process after stress relaxation (point *H*_2_), the reverse transformation progresses from the initiation location at the interfaces of the transformation bands and strain decreases. In the strain holding stage from the point *H*_3_ at a strain of 2% to the point *H*_4_, the reverse transformation progresses and the region of the A-phase expands a little.

As mentioned above, it is thus apparent that stress relaxation and stress recovery are due to the transformation progress under constant strain.

#### 4.1.3. Temperature Change due to Transformation

**Figure 6.**
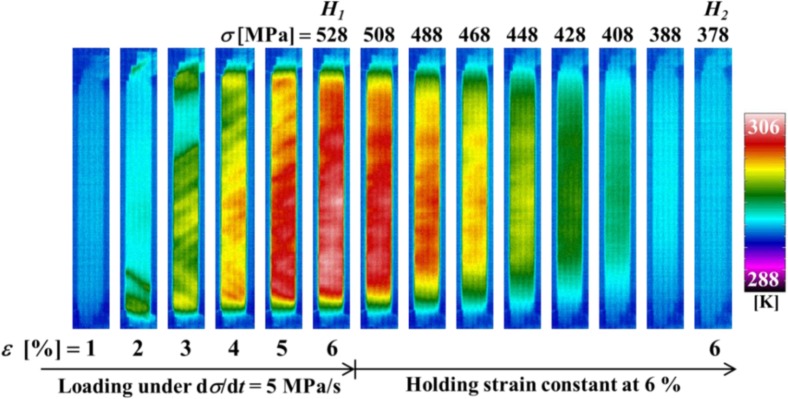
Thermograms of temperature distribution on the specimen surface at various strains ε during loading under a stress rate of 5 MPa/s until a point *H*_1_ (ε_1_ = 6%) and at various stresses σ while holding the strain constant at 6% until point *H*_2_.

**Figure 7.**
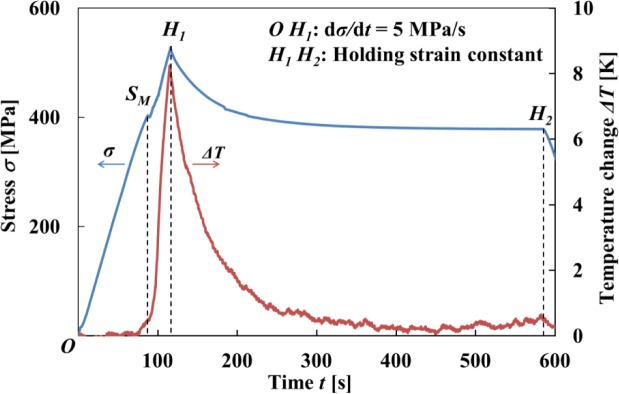
Variation in stress σ and average temperature change Δ*T* on the specimen surface with time *t* during loading under a stress rate of 5 MPa/s until a point *H*_1_ (ε_1_ = 6%) and while holding the strain constant at 6% from point *H*_1_ to point *H*_2_.

**Figure 8.**
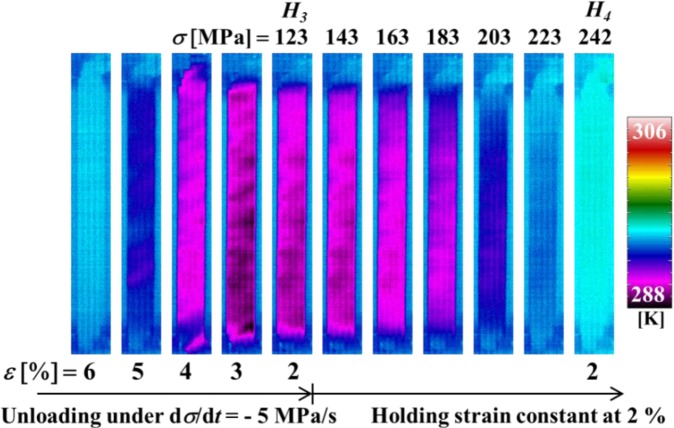
Thermograms of temperature distribution on the specimen surface at various strains ε during unloading under a stress rate of −5 MPa/s till a point *H*_3_ (ε_3_ = 2%) and at various stresses σ while holding the strain constant at 2% until point *H*_4_.

**Figure 9.**
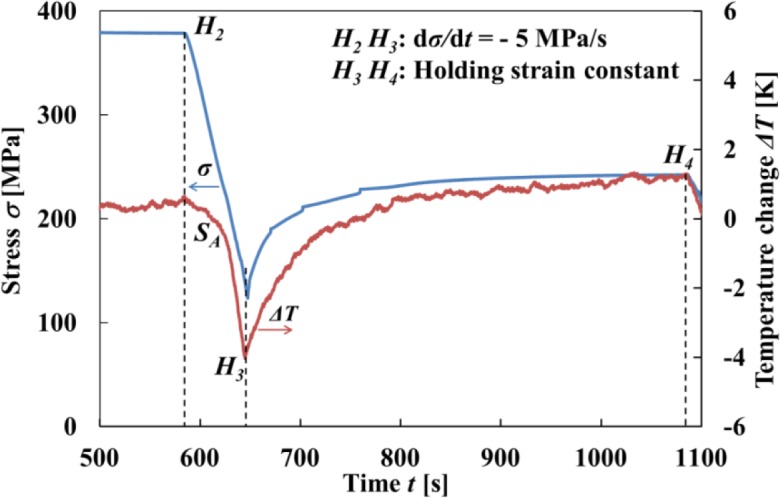
Variation in stress σ and average temperature change Δ*T* on the specimen surface with time *t* during unloading under a stress rate of −5 MPa/s until a point *H*_3_ (ε_3_ = 2%) and while holding the strain constant at 2% from point *H*_3_ to point *H*_4_.

Figure 6 shows the temperature distributions on the specimen surface at various strains during loading and at various stresses while holding the strain constant, obtained by thermography in the test which was carried out under the same conditions as the stress-strain curve shown in Figure 3. Figure 7 shows the relationship of stress σ and temperature change *∆T* between the average temperature on the specimen surface and the atmospheric temperature with time *t* during loading while holding the strain constant. In the same manner, the thermograms of temperature distribution on the specimen surface and the relationship of stress σ and the temperature change *∆T* during unloading while holding the strain constant with time *t* are shown in Figures 8 and 9, respectively. With respect to the thermograms in Figures 6 and 8, in order to express the variation in temperature distribution on the specimen surface while holding the strain constant, the temperature distributions are shown clearly at each stress interval *∆*σ = 20 MPa.

As can be seen in Figures 6 and 7, in the loading process from the MT start point *S_M_* to the point *H*_1_ (ε_1_ = 6%) under a constant stress rate dσ/d*t* = 5 MPa/s, strain rate becomes high as shown in Figure 4 and there exists less time for the heat generated due to the exothermic MT to transfer to the atmospheric air, resulting in an increase in temperature of the specimen. In the strain holding stage from point *H*_1_ to point *H*_2_, the temperature decreases due to the air and the condition for the transformation to progress is satisfied, resulting in progress of the MT. As a result, stress relaxation appears while holding the strain constant.

As can be seen in Figures 8 and 9, in the unloading process from the reverse transformation start point *S_A_* to the point *H*_3_ (ε_2_ = 2%) under a constant stress rate dσ/d*t* = −5 MPa/s, the strain rate becomes high and the temperature decreases due to the endothermic reverse transformation. In the following strain holding stage, the temperature increases due to the air and the condition for the transformation to progress is satisfied. As a result, stress recovery appears due to the reverse transformation while holding the strain constant from point *H*_3_ to point *H*_4_.

As can be seen in Figures 7 and 9, the temperature varies markedly in the early stage while holding the strain constant and thereafter saturates to a certain value.

As mentioned above, the temperature varies due to the progress of the transformation in the strain holding process. It can be stated that stress relaxation and stress recovery appear to be based on this temperature variation.

#### 4.1.4. Condition for Transformation-Induced Stress Relaxation and Stress Recovery to Progress in Stress-Controlled Subloop Loading

**Figure 10.**
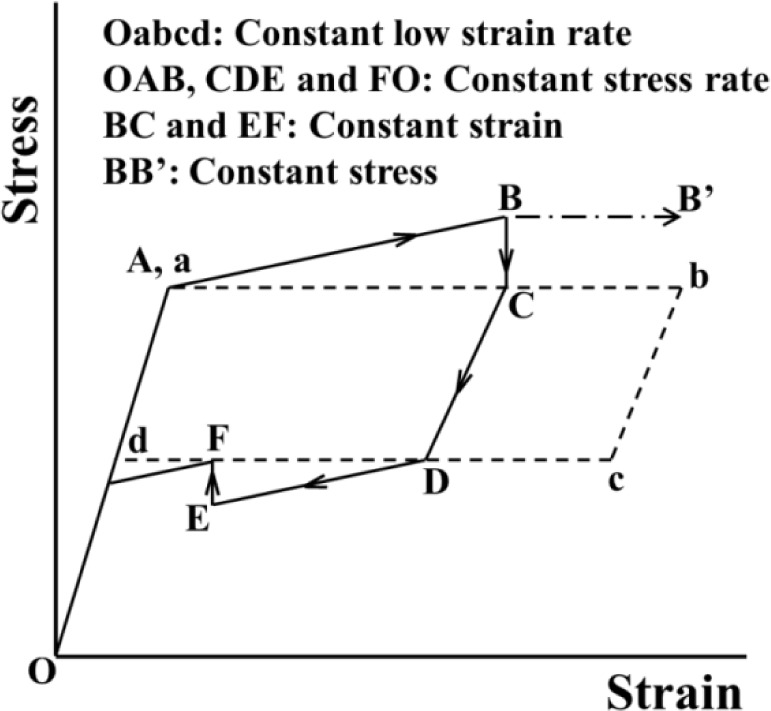
Stress-strain diagrams under (1) the strain-controlled condition (broken line) and (2) the stress-controlled condition (solid line) in SE subloop loading.

**Figure 11.**
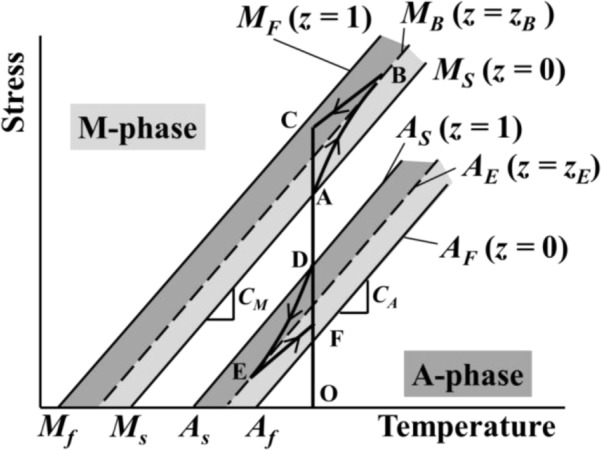
Stress-temperature paths for stress relaxation and stress recovery in the stress-controlled subloop loading (BC and EF: constant strain) on the stress-temperature phase diagram.

In Figure 10, the stress-strain diagram in the SE subloop loading: (1) under a low strain rate is shown by a broken line and (2) by a solid line for the stress relaxation and stress recovery under constant stress rate followed by holding the strain constant and thereafter unloading under a constant stress rate followed by holding the strain constant. The stress-temperature paths for stress relaxation and stress recovery in the stress-controlled subloop loading are shown on the stress-temperature phase diagram in Figure 11. The symbols A, B, C, D, E and F in Figure 10 correspond to those in Figure 11, respectively.

In Figure 11, *z* denotes the volume fraction of the M-phase, and *z_B_* and *z_E_* denote the volume fractions at a point B and a point E, respectively. The symbols *M_S_*, *M_F_*, *A_S_* and *A_F_* denote the start and finish lines of the MT and the reverse transformation with the slopes *C_M_* and *C_A_*, respectively. The broken lines *M_B_* and *A_E_* express the states at the volume fractions *z_B_* and *z_E_*, respectively. The conditions for the transformations to progress are expressed by the following equations [4].

$$\begin{array}{l} d\sigma /dT\geq \;C_{M}:\text{ for}\;dT>0\\ d\sigma /dT\leq \;C_{M}:\text{ for}\;dT<\;0 \end{array}$$ (1)

$$\begin{array}{l} d\sigma /dT\leq \;C_{A}:\text{ for}\;dT>0\\ d\sigma /dT\;\geq C_{A}:\text{ for}\;dT<0 \end{array}$$ (2)

Equation (1) shows the condition for the MT to progress and Equation (2) for the reverse transformation.

In the case of a low strain rate, the MT and reverse transformation progress under a constant stress from a to b and from c to d as shown in Figure 10, respectively. In this case, since the strain rate is low and there exists enough time for the heat generated due to the transformation to transfer to the atmosphere, the temperature change of the specimen is slight and both transformations therefore progress under a constant stress. On the other hand, in the case of the stress-controlled loading and unloading, as shown in Figure 11, since strain rate increases from the transformation start points A (*S_M_*) and D (*S_A_*), the temperature of the specimen changes during AB and DE. As a result, if the strain is held constant thereafter, transformation-induced stress relaxation (during BC) and stress recovery (during EF) appear. In the stress relaxation and stress recovery process, stress changes under constant strain. If it is assumed that the strain change *∆ε* is composed of the elastic strain change *∆*ε*^e^* and the transformation strain change *∆*ε*^t^*, *∆*ε = *∆*ε*^e^* + *∆*ε*^t^* = 0 while holding the strain constant. If the transformation strain change *∆*ε*^t^* appears based on temperature change, the elastic strain change *∆*ε*^e^* = −*∆*ε*^t^*. The relation between elastic strain change *∆*ε*^e^* and stress change *∆*σ is as follows, *∆*ε*^e^* = *∆*σ/*E*, where *E* denotes the elastic modulus. Therefore, stress change *∆*σ = −*E∆*ε*^t^*. As expressed by this equation, if the transformation progresses under constant strain, the stress varies.

If stress is held constant at point B in Figure 10, transformation-induced creep appears (from point B to point B’). The creep appears due to the MT progress. With respect to the creep deformation, since the MT progresses based on a temperature decrease under constant stress, the strain increases [10,13].

### 4.2. Influence of Stress Rate on Stress Relaxation and Stress Recovery

**Figure 12.**
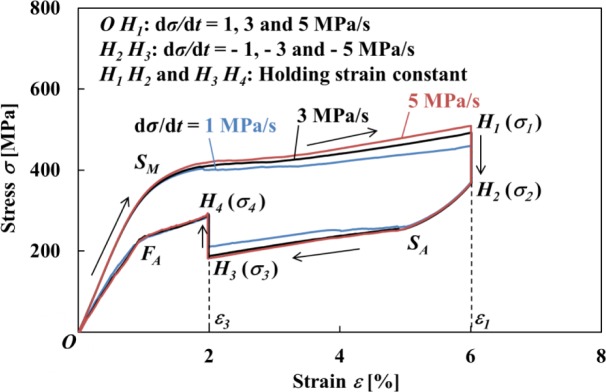
Stress-strain curves obtained by the test under various stress rates dσ/d*t* until point *H*_1_ followed by holding the strain constant at ε_1_ = 6% and thereafter unloading until point *H*_3_ followed by holding the strain constant at ε_3_ = 2%.

The parameters which can be controlled for the thermomechanical properties shown in Figures 10 and 11 are stress rate, holding strain, atmospheric temperature and strain holding time. In the present study, with respect to the constant holding strains ε_1_ = 6% during loading and ε_3_ = 2% during unloading, the constant atmospheric temperature and the strain holding time for the stress to saturate a certain value, the influence of various stress rates dσ/d*t* on stress relaxation and stress recovery was investigated. The stress-strain curves obtained by the test are shown in Figure 12. As can be seen in Figure 12, the higher the stress rate dσ/d*t* in the loading process, then the larger is the temperature rise due to the MT in the upper stress plateau and the higher the stress σ at point *H*_1_. This is caused by the fact that the transformation stress increases in proportion to temperature as shown in Figure 11. On the other hand, the stress σ at the point *H*_2_ does not depend on stress rate and takes almost the same value. As a result, the higher the stress rate, then the larger is the amount of stress relaxation *∆*σ from point *H*_1_ to *H*_2_. In the unloading process, the higher the stress rate, then the larger is the decrease in temperature due to the reverse transformation in the lower stress plateau and the lower the stress σ at the point *H*_3_. On the other hand, the stress σ at the point *H*_4_ does not depend on stress rate and takes almost the same value. As a result, the higher the stress rate, the larger is the amount of stress recovery *∆*σ. The dependence of the characteristics in stress relaxation and stress recovery on the holding strain, atmospheric temperature, strain holding time and stress is a future subject.

As found from the experimental results mentioned above, based on the variation in temperature in the loading and unloading processes, stress relaxation and stress recovery appear due to the progress of the phase transformation under constant strain. Although the change in recovery force of SMA elements due to the temperature change of the atmospheric medium is used in many applications of SMA, the recovery force of SMA does change without temperature change of the atmospheric medium in the stress-controlled subloop loading, accompanied by temperature change due to an increase in strain rate. This point should be noted in the design of SMA elements.

In order to understand the stress relaxation and stress recovery in more detail, it is very important to discuss the deformation properties based on the observation of the microstructure of the material. This subject is for future work.

## 5. Conclusions

The transformation-induced stress relaxation and stress recovery of TiNi SMA tape for SE deformation in stress-controlled subloop loading in the tension test were investigated based on local variation in the transformation band and temperature distribution on the specimen surface observed by microscope and thermography. The influence of the stress rate on stress relaxation and stress recovery is also discussed. The results obtained are summarized as follows.

1. In the loading process under constant stress rate, strain rate increases after the MT starting point and there exists little time for heat generated due to the exothermic MT to transfer to the atmosphere, resulting in temperature rise. In the following strain holding process, the material is cooled by the atmospheric air and the temperature decreases, resulting in stress relaxation due to the MT;
2. In the unloading process under constant stress rate, temperature decreases due to the endothermic reverse transformation after the reverse transformation start point. In the following strain holding process, the material is heated by the atmospheric air and temperature increases, resulting in stress recovery due to the reverse transformation;
3. In the strain holding process, the average temperature on the specimen surface varies markedly in the early stage and thereafter gradually recovers the original temperature. Corresponding to this temperature change, stress relaxation and stress recovery appear markedly in the early stage while holding the strain constant and thereafter stress saturates gradually to a certain value;
4. The behavior of stress relaxation and stress recovery due to the progress of the MT has been confirmed directly by the surface observation of the material;
5. If the stress rate is high until holding the strain constant in the loading and unloading processes, both stress relaxation and stress recovery are large;
6. It is important to take into account stress relaxation and stress recovery in the stress-controlled subloop loading in the design of SMA elements since the force of SMA elements varies due to the temperature variation of the material during loading and unloading even if the atmospheric temperature is kept constant. The fact should be noted that stress relaxation or stress recovery under constant strain occurs without cooling or heating in the SMA elements subjected to the stress-controlled subloop loading.
